# Baseline and Corticotropin-Stimulated Blood Steroid Profiles in Women of Reproductive Age in India: A Cross-Sectional Study and Global Literature Review

**DOI:** 10.7759/cureus.77748

**Published:** 2025-01-20

**Authors:** Shashidhara Revanasiddappa, Melkunte S Dhananjaya, Nimmi Kansal, Anurag Lila, Vijaya Sarathi

**Affiliations:** 1 Endocrinology, Vydehi Institute of Medical Sciences and Research Centre, Bangalore, IND; 2 Clinical Chemistry and Biochemical Genetics, Dr Lal PathLabs Ltd, New Delhi, IND; 3 Endocrinology, Seth Gordhandas Sunderdas Medical College, Mumbai, IND

**Keywords:** acth stimulation, adrenal steroid hormones, blood steroid profile, india, liquid chromatography-tandem mass spectrometry (lc-ms/ms), polycystic ovarian syndrome, reference ranges, reproductive-age women

## Abstract

Introduction: Corticotropin (adrenocorticotropic hormone (ACTH))-stimulated blood steroid profile is useful in the diagnosis of rare adrenal disorders but their utility is limited due to lack of assay- and population-specific reference ranges. Hence, we conducted this study to establish the reference ranges for basal and ACTH-stimulated blood steroid profiles in Indian women of reproductive age.

Methods: This cross-sectional study included 30 apparently healthy female volunteers aged 18-40 years. Blood samples were collected during the early follicular phase of the menstrual cycle at baseline and 60 minutes after intramuscular administration of 250 µg of ACTH 1-24. Serum steroid levels were quantified using liquid chromatography-tandem mass spectrometry (LC-MS/MS).

Results: After ACTH stimulation, 11-deoxycorticosterone (24.8±19.4 ng/dl from 2.44±0.8 ng/dl, 10.16-fold), corticosterone (from 226.0±184.0 to 2159.0±779.0 ng/dl, 9.55-fold) and 11-deoxycortisol (from 47.4±52.4 to 344.7±246.0 ng/dl, 7.28-fold) demonstrated greater than seven-fold elevation whereas 17α-hydroxyprogesterone (from 38.0±39.0 ng/dl to 129.0±75.0 ng/dl, 3.39-fold), dehydroepiandrosterone (from 340.0±221.8 to 998.0±580 ng/dl, 2.94-fold), cortisol (from 10.90±3.80 to 24.72±4.46 µg/dl, 2.27-fold) and aldosterone (from 6.65±5.19 to 13.88±8.11 ng/dl, 2.09-fold) showed 2-3.5-fold elevation.

Conclusions: This study presents reference ranges for baseline and ACTH-stimulated steroid panels in Asian Indian women of reproductive age and summarises the available blood steroid profiles for reproductive-age women from the literature.

## Introduction

The prevalence of polycystic ovarian syndrome (PCOS) in Indian women ranges from 3.7% to 22.5% [1]. It poses a diagnostic challenge, requiring the exclusion of various forms of hyperandrogenism, including rarer forms of congenital adrenal hyperplasia (CAH) and glucocorticoid resistance syndrome [2-4]. However, evaluation for secondary causes of PCOS primarily focuses on excluding 21-hydroxylase deficiency (21-OHD) through immunoassay-based measurements of basal ± adrenocorticotropic hormone (ACTH)-stimulated serum 17-hydroxyprogesterone (17-OHP). This approach may miss or misdiagnose rarer forms of CAH [3-5].

The steroid profile includes the simultaneous measurement of several adrenal steroid hormones by a more accurate assay, liquid chromatography-tandem mass spectrometry (LC-MS/MS); hence, it is a useful tool to exclude non-PCOS causes of hyperandrogenism in women. However, the reliable utility of blood steroid profiling requires assays with population-specific reference ranges, and reference ranges for ACTH-stimulated steroid profiling are lacking for the Indian population. This hinders the interpretation of the ACTH-stimulated steroid profile. A few studies from Europe have reported the reference ranges for the ACTH-stimulated steroid profile, but the majority of them include both men and women with no female-specific reference ranges [6-8].

Blood steroid profile has been recently introduced in India [9]. In a recent study, we provided reference ranges for the acton prolongatum (long-acting porcine sequence ACTH)-stimulated steroid profile using a commercially available kit (PerkinElmer, Inc., Shelton, Connecticut, United States) in apparently healthy Asian Indian women of reproductive age [10]. In the present study, we report reference ranges for baseline and ACTH 1-24 (tetracosactide)-stimulated steroid levels for an updated steroid panel (Chromsystems Instruments & Chemicals GmbH, Gräfelfing, Germany) in apparently healthy Asian Indian women of reproductive age.

## Materials and methods

This cross-sectional study was conducted at a tertiary healthcare center, Vydehi Institute of Medical Sciences and Research Center, in Bangalore, India. The study was approved by the Vydehi Institutional Ethics Committee (approval number: VIEC/2022/APP/003) and conducted following the Declaration of Helsinki. Written informed consent was obtained from all study participants.

Participants and sample size

The study included 30 apparently healthy Asian Indian women of reproductive age (18-40 years). Participants who were apparently healthy and exhibited regular menstrual cycles with no clinical features of hyperandrogenism were included. Participants with pregnancy or lactation, oral contraceptive pill usage, or acute or chronic systemic illness were excluded. The participants were recruited from the community. The sample size of 30, the threshold at which the central limit theorem applies, was pragmatically determined to ensure feasibility, given the limited acceptability of the ACTH stimulation test among participants (healthy volunteers).

Data collection

After providing written consent, participants underwent a thorough evaluation of their medical history, including an assessment of menstrual cycles, symptoms of hyperandrogenism, and a review of family history for atypical genitalia, and features of hyperandrogenism. Comprehensive physical examinations, including anthropometric measurements (height, weight, BMI) and assessments for hyperandrogenism features such as acne and the modified Ferriman-Gallwey score, were conducted.

Participants were evaluated during the follicular phase (the first seven days of the menstrual cycle). Fasting (>8 hours) blood samples were collected at 8:00 am for the basal steroid panel, fasting plasma glucose, lipid profile, and serum albumin. Subsequently, 250 µg of ACTH 1-24 (Syntropac; Sun Pharma Ltd., Vadodara, Gujarat, India) was administered intramuscularly, and another blood sample was collected 60 minutes later. The samples were centrifuged, and the separated serum was sent to Dr Lal PathLabs, New Delhi, India, for the estimation of blood steroid levels (steroid panel 3). This panel included 11-deoxycorticosterone, 11-deoxycortisol, 17-OHP, 21-deoxycortisol, aldosterone, androstenedione, corticosterone, cortisol, cortisone, dehydroepiandrosterone (DHEA), dehydroepiandrosterone sulfate (DHEAS), progesterone, and testosterone.

Quantification of steroid levels in this study utilized the ExionLC™ AD series autosampler (Danaher Corporation, Washington, D.C., United States) coupled with the SCIEX Triple Quad 4500 LC-MS/MS System (Danaher Corporation). Reagents, including an in-vitro diagnostics (IVD)-certified kit, MassChrom® Steroids (Chromsystems Instruments & Chemicals GmbH, Gräfelfing, Germany), as well as internal standards, calibrators, analytical columns, and controls (Chromsystems Instruments & Chemicals GmbH). Given that steroids are protein-bound, a solid-phase extraction method was employed for sample preparation. The addition of an extraction buffer initiated the removal of protein interactions, allowing the determination of both bound and free steroids as total steroids. Following extraction, internal standards were added, with each analyte paired with its isotopically labeled internal standard to ensure consistent and reliable quantitative results.

Subsequently, the well plate underwent a wash with a buffer to eliminate unbound compounds, followed by the addition of an elution buffer and nitrogen evaporation at 50°C to achieve dryness. The dried plate was then subjected to reconstitution with a buffer. After shaking the plate, the supernatant was collected into auto-sampler vials, and the eluate was injected into the LC-MS/MS system. Steroid hormones underwent separation by an analytical column and were quantified based on their specific multiple reaction monitoring (MRM) transitions. This robust method ensured precise and accurate quantification of steroid hormones in the samples.

Statistical analysis

All data were entered and analyzed using IBM SPSS Statistics for Windows, Version 22.0 (Released 2013; IBM Corp., Armonk, New York, United States). Reference ranges were derived using the nonparametric method (minimum-maximum), as the sample size was less than 40 [11]. A paired t-test compared steroid levels at baseline and after ACTH stimulation. A p-value < 0.05 was considered statistically significant for all analyses.

## Results

The demographic, anthropometric, and metabolic characteristics of the participants are summarized in Table 1. Blood samples for the steroid profile were collected an average of 3.76 ± 1.22 days after menstruation.

**Table 1:** Demographic, anthropometric, and metabolic characteristics of the participants (N=30) HDL: high-density lipoprotein; LDL: low-density lipoprotein

Parameters	Observed values, mean±SD
Age (years)	25.5±2.4
Weight (cm)	55.82±3.46
Height (cm)	162.14±5.96
Body mass index (kg/m2)	21.23±1.89
Waist circumference (cm)	78.21±2.67
Serum albumin (g/dl)	4.06±0.58
Fasting plasma glucose (mg/dl)	85.4±7.18
Fasting serum insulin (µIU/ml)	9.12±3.29
Serum total cholesterol (mg/dl)	132.3±13.63
Serum triglycerides (mg/dl)	93.9±15.31
Serum HDL-cholesterol (mg/dl)	49.8±5.65
Serum LDL-cholesterol (mg/dl)	80.81±11.47

Baseline and ACTH-stimulated steroid levels are presented in Table 2. The largest increment (10.16-fold) was observed in 11-DOC (from 2.44 ± 0.8 to 24.8 ± 19.4 ng/dl). Corticosterone levels increased from 226.0 ± 184.0 to 2159.0 ± 779.0 ng/dL (9.55-fold), and 11-deoxycortisol rose from 47.4 ± 52.4 to 344.7 ± 246.0 ng/dL (7.28-fold), both demonstrating greater than five-fold elevations. Moderate elevations (2-5-fold) were observed in 17α-hydroxyprogesterone (from 38.0 ± 39.0 to 129.0 ± 75.0 ng/dL, 3.39-fold), dehydroepiandrosterone (DHEA) (from 340.0 ± 221.8 to 998.0 ± 580.0 ng/dL, 2.94-fold), cortisol (from 10.90 ± 3.80 to 24.72 ± 4.46 µg/dL, 2.27-fold), and aldosterone (from 6.65 ± 5.19 to 13.88 ± 8.11 ng/dL, 2.09-fold). Smaller increases (<2-fold) were noted in androstenedione (from 103.74 ± 501.28 to 176.27 ± 67.02 ng/dL, 1.7-fold), total testosterone (from 22.6 ± 10.24 to 26.4 ± 9.26 ng/dL, 1.17-fold), and DHEAS (from 134.8 ± 782.71 to 148.81 ± 90.35 µg/dL, 1.11-fold). No significant changes were observed in cortisone (from 1.98 ± 4.77 to 1.94 ± 0.35 µg/dL) or 21-deoxycortisol (from 5.67 ± 4.6 to 9.23 ± 8.0 ng/dL). The baseline cortisol to cortisone ratio was 5.58 ± 1.71 (3.37- 11.03) which rose to 13.22 ± 2.35 (7.92 - 18.95) after ACTH stimulation.

**Table 2:** Basal and ACTH-stimulated adrenal steroid levels measured by LC-MS/MS (N=30) *p-value: < 0.05 DHEA: dehydroepiandrosterone; DHEAS: dehydroepiandrosterone sulphate; ACTH: adrenocorticotropic hormone; LC-MS/MS: liquid chromatography-tandem mass spectrometry

Hormone	Baseline mean±SD (range)	ACTH stimulated mean±SD (range)	X-fold elevation mean ± SD
11-Deoxycorticosterone (ng/dl)	2.44±0.8 (1.0-6.0)	24.8±19.4 (3.0-86.0)*	10.16±24.25
11-Deoxycortisol (ng/dl)	47.4±52.4 (4.0-219.0)	344.7±246.0 (38.0-764.0)*	7.28±4.69
17α-Hydroxyprogesterone (ng/dl)	38.0±39.0 (9.0-223.0)	129.0±75.0 (40.0-312.0)*	3.39±1.92
21-Deoxycortisol (ng/dl)	5.60±4.60 (<2.7-15.0)	9.2±8.0 (<0.27-33.0)	1.64±1.73
Aldosterone (ng/dl)	6.65±5.19 (1.40-20.0)	13.88±8.11 (4.20-30.50)*	2.09±1.56
Androstenedione (ng/dl)	103.74±50.12 (36.0-236.0)	176.27±67.02 (85.40-344.00)	1.70±1.33
Corticosterone (ng/dl)	226.0±184.0 (48.0-643.0)	2159.0±779.0 (56.0-3550.0)*	9.55±4.23
Cortisol (µg/dl)	10.90±3.80 (6.30-22.40)	24.72±4.46 (15.60-33.70)*	2.27±1.17
Cortisone (µg/dl)	1.98±0.47	1.94±0.35	0.97±0.74
DHEA (ng/dl)	340.0±221.8 (80.0-727.0)	998.0±580 (224.0-2520.0)*	2.94±2.62
DHEAS (µg/dl)	134.68±78.27 (30.7-314.0)	148.80±90.35 (35.50-401)*	1.11±1.15
Progesterone (ng/dl)	6.29±4.60 (1.0-17.0)	32.89±21.3 (5.8-79)*	5.24±4.63
Total testosterone (ng/dl)	22.6±10.24 (8.0-47.0)	26.4±9.26 (12.0-55)*	1.17±0.90

## Discussion

Population-specific reference ranges for baseline and ACTH-stimulated steroid panels in Indian reproductive-aged women were presented in this study. Several studies have provided reference ranges for baseline steroid profiles by LC-MS/MS in reproductive-aged women (Table 3) [7,8,12-18].

**Table 3:** Basal steroid hormone levels in premenopausal women in global literature * median (IQR), ^ range 17OHP: 17-hydroxy progesterone; DHEA: dehydroepiandrosterone; DHEAS: dehydroepiandrosterone sulphate

Study, year	Altinkilic et al., 2023 [12]	Bachelot et al., 2023 [13]	Bachelot et al., 2023 [13]	Deng et al., 2017 [14]	Fanelli et al., 2011 [15]	Kulle et al., 2013 [16]	Tennilä et al., 2021 [17]	Handelsman et al., 2017 [18]
Country	Switzerland, Turkey	France	France	China	Italy	Germany	Finland	Australia
Remarks		Training set	Test set	BMI< 25 kg/m2				
Total sample size, n	42	19	72	203	134	86	21	45
Females, n	42	19	72	203	134	86	21	45
Age (years)	27.5 (23.8-33.3)*	NA	NA	26 (24-32)*	18-54^	16-40^	18.1 (17.9-18.3)*	18-45^
	Median (IQR)	Median (IQR)	Median (IQR)	Median (IQR)	Median (95% CI)	Median (95% CI)	Median (IQR)	Mean (SEM)
11-deoxycorticosterone (ng/dl)	4.90 (2.01-7.21)	4 (3-5)	5 (4-5)	4 (2.8-6)		8.0 (6.0-54)		
11-deoxycortisol (ng/dl)	25.67 (11.25-42.4)			25 (15-39)	23.90 (<108.1)	18.0 (9.0-106)		
17OHP (ng/dl)	68.95 (41.25-129.24)	100 (50-149)	93 (69-116)	27 (11.60-71)	57.80 (15.2-226)	30.0 (6.0-85)	62.31 (33.75-89.14)	80 (10)
21-deoxycortisol (ng/dl)		2 (1-3)	1.0 (1-2)			6.0 (4.0-35)		
Aldosterone (ng/dl)	9.23 (4.90-14.71)	12 (7-17)	9 (7-11)	3.7 (2.40-5.9)				
Androstenedione (ng/dl)	155.79 (104.72-244.35)	85 (63-108)	118 (101-136)	183 (136-226)	74.80 (27.7-163.8)		192.71 (145.69-234.55)	100 (5.0)
Corticosterone (ng/dl)	271.19 (143.38-473.42)	289 (201-377)	394 (313-476)	155 (96-267)	262 (62.-1185)	99.0 (9.0-702)		
Cortisol (µg/dl)	10.97 (8.06-15.79)	8.36 (7.09-9.64)	10.74 (9.43-11.51)	7.35 (5.31-10.2)	10.15 (4.74-19.97)	9.78 (5.01-29.36)		11.1 (0.7)
Cortisone (µg/dl)	2.1 (1.69-2.68)	1.84 (1.6-2.07)	1.94 (1.84-2.04)	2.93 (2.30-3.85)		1.84 (0.29-3.23)		
DHEA (ng/dl)	533.43 (306.67-747.79)	496 (383-610)	780 (641-919)		509 (119-1893)		983.78 (802.03-1373.26)	425 (33)
DHEAS (µg/dl)	198.45 (124.52-276.42)			99.90 (72.85-139.25)			132.50 (94.20-184.26)	
Progesterone (ng/dl)	25.96 (8.078-527.08)	401 (113-690)	315 (160-470)	47.6 (12.1-18)	20.3 (1781.60)	11.0(4.0-491.0)	10.01 (7.73-31.75)	390 (100)
Testosterone (ng/dl)	33.46 (23.36-49.33)	25 (20-29)	36 (31-40)	32.8 (26.0-41.6)	24.8 (10.4-45.4)		46.44 (31.44-52.50)	23.0 (2.0)

Reference ranges are also available for specific populations, including overweight [14], obese [14], and postmenopausal women [14], as well as for the specific phases of the menstrual cycle, such as the follicular phase (days 1-10 of the menstrual cycle) (Table 4) [15,19].

**Table 4:** Basal steroid profile in special female populations from the literature * median (IQR), ^ range 17OHP: 17-hydroxy progesterone; DHEA: dehydroepiandrosterone; DHEAS: dehydroepiandrosterone sulphate

Study, year	Deng et al., 2017 [14]	Deng et al., 2017 [14]	Fanelli et al., 2011 [15]	Fanelli et al., 2011 [15]	De Dalmazi et al., 2015 [19]
Country	China	China	Italy	Italy	Italy
Remarks	BMI: 25-30 kg/m^2^	BMI: >30 kg/m^2^	Follicular	Postmenopausal	Postmenopausal
Total	32	131	51	65	128
Females	32	131	51	65	128
Age (Years)	25.0 (20.0-29.0)*	25.0 (21.0-30.0)*	18-54^	45-86^	60.3 (58.5-62.1)*
	Medain (IQR)	Medain (IQR)	Median (95% CI)	Median (95% CI)	Mean ((95% CI)
11-deoxycorticosterone (ng/dl)	4.10 (2.90-5.40)	3.6 (2.30-6.50)			8 (7.8-8.2)
11-deoxycortisol (ng/dl)	18.70 (9.40-38.40)	32.20 (14.30-53.50)	24.90 (<134.50)	28.4 (8.20-83.80)	33 (30-37)
17OHP (ng/dl)	27.3 (9.70-77.20)	18.40 (7.40-47.50)	41.10 (16.10-94.70)	20.90 (<52.70)	32 (28-36)
Aldosterone (ng/dl)	3.8 (2.80-5.90)	3.70 (2.10-6.40)			
Androstenedione (ng/dl)	195.0 (151.80-229.80)	186.0 (143.0-261.0)	72.70 (30.80-160.20)	29.90 (9.50-77.30)	35 (31-38)
Corticosterone (ng/dl)	158.0 (108.8-209.0)	175.0 (95.30-307.0)	289.0 (45.0-1194.0)	274.0 (68.0-854.0)	314 (275-358)
Cortisol (µg/dl)	6.8 (5.54-8.89)	8.16 (5.58-11.7)	11.31 (4.05-19.98)	11.47 (5.67-18.04)	11.61 (10.95-12.3)
Cortisone (µg/dl)	3.13 (2.25-3.93)	2.97 (2.15-4.10)			
DHEA (ng/dl)			568.0 (203.0-2704.0)	245.0 (80.0-660.0)	252 (225-282)
DHEAS (µg/dl)	91.8 (73.70-122.0)	93.0 (71.30-129.75)			
Progesterone (ng/dl)	21.3 (7.0-331.5)	21.8 (7.0-97.7)	9.30 (<167.30)	4.90 (<8.0)	8.9 (6.9-11.4)
Testosterone (ng/dl)	33.8 (26.10-44.10)	34.5 (24.8-44.6)	24.80 (11.6-43.10)	14.70 (7.70-39.20)	18 (16-19)

In the current study, blood samples were collected during the early follicular phase (the first seven days of the menstrual cycle). While this focus on a specific menstrual phase enhances the precision of the reference ranges despite the small sample size, it limits their utility to the follicular phase of the menstrual cycle. Notably, luteal phase-specific reference ranges are not yet available, and developing such ranges could significantly expand the applicability of steroid profiling.

Variability across studies is noted in baseline median levels of all steroid hormones in premenopausal women including 11-DOC (2.88-8.0 ng/dl), 11-deoxycortisol (18.1-32.2 ng/dl), aldosterone (3.7-12 ng/dl), androstenedione (72.7-195 ng/dl), 17OHP (18.4-80 ng/dl), corticosterone (99-394 ng/dl), cortisol (6.8-11.7 µg/dl), cortisone (1.71-3.13 µg/dl), DHEA (425-983.78 ng/dl), DHEAS (91.8-198.45 µg/dl) and total testosterone (24.8-46.44 ng/dl) (Table 3). Steroid hormone levels in the present study were comparable to those reported in previous studies, except for DHEA, which was notably lower (340.0 ± 221.8 ng/dL). This observation warrants further investigation. Additionally, levels of androstenedione, testosterone, DHEA, and DHEAS were lower in postmenopausal women, emphasizing the need for specific reference ranges for this population.

A few previous studies have reported basal and ACTH-stimulated blood steroid profiles in healthy controls (Table 5) [7,8,20]. The majority of these studies included both men and women, with female-specific reference ranges reported in only one study [8]. We previously reported reference ranges for acton prolongatum-stimulated steroid profiles in reproductive-age women [10], and the present study is the second to report both basal and ACTH-stimulated blood steroid profiles exclusively in female participants. In comparison to our previous study [10], serum corticosterone levels were higher in the present study. This difference was attributed to assay-related differences rather than the differences in the form of ACTH used.

**Table 5:** Basal and corticotropin-stimulated steroid profiles from the literature 17OHP: 17-hydroxy progesterone, ACTH: adrenocorticotropic hormone, DHEA: dehydroepiandrosterone, DHEAS: dehydroepiandrosterone sulphate

Study, year	Kulle, 2015 [7]	Kulle, 2015 [7]	Kulle, 2015 [7]	Lindner, 2017 [8]	Lindner, 2017 [8]	Ueland, 2022 [20]	Ueland, 2022 [20]
	Basal	ACTH-30 min	ACTH-60 min	Basal	ACTH	Basal	ACTH
Country	Germany	Germany	Germany	Germany	Germany	Norway	Norway
Total study participants, n	44	44	44	36	36	66	66
Females, n	19	19	19	26	26	66	66
Age (years), range	8-58	8-58	8-58	22-58	22-58	23-68	23-68
	Median (90% CI)	Median (90% CI)	Median (90% CI)	Median (range)	Median (range)	Median (range)	Median (range)
11-deoxycorticosterone (ng/dl)	3 (1-13)	12 (3-29)	17 (3-31)	3.4 (2.2-8.8)	19.1 (3.7-11.5)	3.74 (2.04-10.96)	15.57 (6.05-49.03)
11-deoxycortisol (ng/dl)	10 (3-84)	44 (3-152)	62(3-163)	18.1 (10.8-37.4)	75.8 (24.1-437)	15.28 (7.49-49.03)	54.79 (22.49-147.09)
17OHP (ng/dl)	76 (4-187)	104 (46-279)	112 (42-224)	30.5 (5.8-242)	112 (44.2-292)	52.87 (10.90-152.0)	105.74 (39.65-280.89)
21-deoxycortisol (ng/dl)	3 (3-14)	3 (3-39)	3 (3-60)			<7.21 (<7.21-<7.21)	12.97 (7.49-49.03)
Androstenedione (ng/dl)	78.42 (15.48-152.36)	90.52 (23.41-171.11)	114.07 (28.98-190.09)			95.17 (34.61—245.15)	132.67 (46.14-317.26)
Corticosterone (ng/dl)	306 (15-1622)	1767 (772-3024)	2484(1031-4144)	177 (68.4-808)	3240 (1730-4680)	224.96 (40.37-1269.04)	2278.51 (1182.52-4182.09)
Cortisol (µg/dl)	16.19 (5.31-31.99)	21.19 (11.81-36.95)	24 (13.6-36.7)	9.26 (3.24-21.3)	26.7 (18.1-40.7)		
Cortisone (µg/dl)	2.25 (1.23-3.27)	1.99 (1.15-2.60)	1.73 (1.02-2.26)	1.77 (1.12-2.94)	1.78 (1.14-2.34)	0.28 (0.90-3.29)	1.59 (0.65-2.93)
DHEA (ng/dl)						374.94 (100.94-2163.35)	1009.47(248.04-3316.83)
Progesterone (ng/dl)	3 (3-38)	20 (3-52)	25 (8-60)				
Testosterone (ng/dl)	30.53 (7.37-290.77)	26.81 (4.88-75.52)	30.04 (10.56-433.20)			23.07 (7.21-51.91)	

In the first published ACTH-stimulated steroid profile by Holst et al., 11-deoxycortisol and cortisol levels were relatively higher, whereas aldosterone, androstenedione, and DHEA levels were relatively lower [21]. Studies from Germany [7,8,22] and Norway [20] have reported lower 11-deoxycortisol levels, while Indian studies [10], including the present one, and a Brazilian study involving older participants [23], observed intermediate levels. These findings may reflect ethnicity-related differences. 

The upper limit of normal for ACTH-stimulated 17-OHP ranged from 112 to 620.1 ng/dL, while the lower limit of normal for ACTH-stimulated cortisol was below 18 µg/dL in approximately half of the studies (Table 5 and Table 6) [7,8,10,20,23]. These findings highlight the need to revise these cut-offs to enhance sensitivity for diagnosing the non-classic form of 21α-hydroxylase deficiency and reduce the risk of overdiagnosing adrenal insufficiency, respectively.

**Table 6:** Corticotropin-stimulated steroid profile * age expressed as mean±SD, 17OHP: 17-hydroxy progesterone, ACTH: adrenocorticotropic hormone, DHEA: dehydroepiandrosterone, DHEAS: dehydroepiandrosterone sulphate, N: normal, SAI: suspected adrenal insufficiency

Study, year	Holst, 2007 [21]	Sarathi, 2022 [10]	Briegel, 2022 [23]
Country	USA	India	Germany
Total	61 (21 N + 40 SAI)	32	20
Females	38 (12 N + 26 SAI)	32	10
Age (Years)*	33±9	22.19±4.36	33.1±9.9
Mode of data expression	Mean±SD	Median (range)	Median (IQR)
11-deoxycorticosterone (ng/dl)			20.0 (10-30)
11-deoxycortisol (ng/dl)	670±290	287.2 (74.41-530.61)	100.0 (50-110)
17OHP (ng/dl)	162±163	99.72 (47.21-344.71)	140 (100-200)
21-deoxycortisol (ng/dl)			
Aldosterone (ng/dl)	9.53±5.58	18.9 (3.59-42.30)	
Androstenedione (ng/dl)	78±50	136.68 (52.85-190.03)	
Corticosterone (ng/dl)		728.04 (118.74-1708.2)	3030.0 (2740.0-3440.0)
Cortisol (µg/dl)	31.07±7.85	22.65 (14.3-37.21)	25.86 (24.49-26.93)
Cortisone (µg/dl)			1.95 (1.76-2.05)
DHEA (ng/dl)	553±429	764.25 (306.60-1762.83)	
DHEAS (µg/dl)	141±152	234.09 (89.60-387.07)	
Progesterone (ng/dl)		16 (8-43)	
Testosterone (ng/dl)		34.37 (17.75-47.49)	

The greatest response to ACTH stimulation was shown by 11-deoxycortisol, corticosterone, and 11-DOC, with elevations ranging from 7.28 to 10.16-fold from baseline. In previous studies, only corticosterone exhibited a 10-fold elevation, while 11-DOC and 11-deoxycortisol showed around a five-fold increase [7,8,20]. The relatively greater elevation of the latter two steroids in our study may suggest a reduced sensitivity of the 11β-hydroxylase enzyme to acute pharmacological doses of ACTH in Asian Indians, warranting further investigation. In an adrenal cell study by Xing et al., cortisol showed the greatest increase (63-fold) following ACTH stimulation, followed by corticosterone (37-fold), 17OH-pregnenolone (32.2-fold), 11-deoxycortisol (23.1-fold), 17OHP (15.6-fold), DHEA (17.6-fold), and 11DOC (12.8-fold) [24]. The relative increases in some adrenal steroids in their in vitro study were not consistent with our findings. Notably, a German study involving individuals with bilateral adrenal hyperplasia found that while the contribution of cortisol to steroid levels in the adrenal vein remained comparable before and after ACTH stimulation, the contributions of 11-deoxycortisol, corticosterone, and 11-DOC increased by approximately 2.5-3.5-fold, which aligned with their respective changes in peripheral veins [25]. These observations are in accordance with those from our study. 

Interestingly, aldosterone which is normally under little to no influence of ACTH under physiological conditions, showed a 1.68-fold increase after ACTH stimulation. Similar observations have been reported previously [23,25]. However, this increase is acute, and aldosterone levels decrease with continued ACTH infusion [24]. ACTH-stimulated aldosterone has demonstrated diagnostic utility in distinguishing primary from secondary adrenal insufficiency, as well as in the diagnosis of primary aldosteronism [26-28]. The increase in DHEA after ACTH stimulation was 2.43-fold, while the increase in DHEAS was marginal, suggesting that ACTH does not have an acute effect on the sulfation of DHEA. Similarly, no significant increase was observed in cortisone levels, indicating that ACTH stimulation does not acutely affect the activity of 11β-hydroxysteroid dehydrogenase 1.

Limitations

A small sample size limited the study. However, this was a common limitation in most previous studies, as many healthy volunteers are hesitant to undergo invasive tests like the ACTH stimulation test [7,8,10,20,23]. Notably, reference ranges in this study were derived using the nonparametric method (minimum-maximum), which is the most accurate for estimating reference intervals when the sample size is less than 40 [11]. Interestingly, unlike the majority of previous studies [7,8,20,23], our sample was more homogeneous. However, this may restrict the reference ranges to young adult women of reproductive age in the follicular phase, limiting their generalizability. Particularly, excluding adolescents, the age group in which ACTH stimulation test is more often performed to exclude secondary causes of PCOS, is an important limitation. In some studies, a few healthy volunteers exhibited unexpectedly high testosterone or 17-OHP levels [8,22], highlighting the need for careful selection of participants. The stringent criteria used in our study to exclude women with irregular menstrual cycles and clinical hyperandrogenism helped ensure appropriate participant selection. It is worth noting that while we did not perform a pelvic ultrasound to exclude polycystic ovarian morphology (PCOM) in our participants, the lack of the other two diagnostic criteria excludes PCOS irrespective of the presence of PCOM.

## Conclusions

This study reports the reference ranges for baseline and ACTH-stimulated serum steroid profiles in Asian Indian women of reproductive age. The reference ranges for baseline and ACTH-stimulated blood steroid profiles were also summarized from the literature, with a special focus on women of reproductive age. These reference ranges help in the accurate diagnosis of adrenal disorders. A relatively greater increase in 11-DOC and 11-deoxycortisol in Asian Indian women may suggest a lesser acute effect of ACTH on 11β-hydroxylase activity in them. Further studies are warranted in this regard.
